# Pointing Gesture Based User Interaction of Tool Supported Brainstorming Meetings

**DOI:** 10.1007/978-3-030-58805-2_3

**Published:** 2020-08-12

**Authors:** Naina Dhingra, Reinhard Koutny, Sebastian Günther, Klaus Miesenberger, Max Mühlhäuser, Andreas Kunz

**Affiliations:** 8 Institute Integriert Studieren, JKU Linz, Linz, Austria; 9 Jack Baskin School of Engineering, UC Santa Cruz, Santa Cruz, CA USA; 10 Dipartimento di Meccanica, Politecnico di Milano, Milan, Italy; 11 Support Centre for Students with Special Needs, Masaryk University Brno, Brno, Czech Republic; 12 Innovation Center Virtual Reality, ETH Zurich, Zurich, Switzerland; 13 Institut Integriert Studieren, Johannes Kepler University, Linz, Austria; 14 Technische Universität Darmstadt, Darmstadt, Germany

**Keywords:** Brainstorming tool, Web application, Android application, Pointing gesture, Robot operating system, Kinect sensor, OpenPtrack, Localization, Recognition, Non-verbal communication

## Abstract

This paper presents a brainstorming tool combined with pointing gestures to improve the brainstorming meeting experience for blind and visually impaired people (BVIP). In brainstorming meetings, BVIPs are not able to participate in the conversation as well as sighted users because of the unavailability of supporting tools for understanding the explicit and implicit meaning of the non-verbal communication (NVC). Therefore, the proposed system assists BVIP in interpreting pointing gestures which play an important role in non-verbal communication. Our system will help BVIP to access the contents of a Metaplan card, a team member in the brainstorming meeting is referring to by pointing. The prototype of our system shows that targets on the screen a user is pointing at can be detected with 80% accuracy.

## Introduction

Non-verbal communication plays an important role in team meetings, in which we use gestures along with speech to convey the full meaning of our ideas. Usually, those gestures are based on our inherited cultures, language we speak, etc. However, this non-verbal communication (NVC) is not accessible to blind and visually impaired people (BVIP) without additional aid. Thus, they are unable to participate in the meetings to a full extent. To better integrate BVIP in such meetings, we need to provide them with external aids that are able to capture and transfer the spatial information of artifacts as well as referring gestures and other non-verbal communication elements by sighted users.

Brainstorming meetings are used in many areas of business and academia, such as medical diagnostics, scientific research, spin-offs, military operations, etc. Considering the wide use of brainstorming meetings, there is a need to build an autonomous system to help BVIP work independently in those meetings. Otherwise, it is very difficult for them to understand the full meaning of the conversation, mainly due to the non-verbal communication.

NVC in brainstorming meetings includes several kinds of gestures performed by the participants, such as nodding, shaking the head, head orientation, pointing gestures, sign language, eye contact, blinking of eyes, pointing with eyes, etc. Thus, the information flow in a team meeting is not simply based on generated artifacts and on spoken explanations, but it is in particular a manifold of NVCs that could carry up to 55% of the overall information [13]. These gestures refer to the 3D information space they are performed in.

Spatial aspects of brainstorming meetings also play a vital role in understanding and determining pointing gestures performed by the participants of a meeting. Most people tend to give an egocentric relative position of the objects in the meeting room when referring to them. Some of the spatial artifacts which are to be considered are whiteboards, items on the whiteboards, etc. For this paper, we developed a Metaplan brainstorming tool which is the basis of our spatial artifacts.

Thus, the goal is to transfer NVC elements to BVIP, and more particular pointing gestures that refer to artifacts in the 3D information space. For this, we use OpenPtrack along with robot operating system (ROS) [18] to detect the pointing direction of a user with regard to artifacts in a common work space. We have also developed a brainstorming tool which has a web interface (the “Moderator” interface) and android application for the digital interaction between the members of the brainstorming meeting. The content of the corresponding artifact could then be output on a blind user interface such as braille.

This paper is structured as follows: Related work is discussed in Sect. 2, while the methodology is described in Sect. 3. The experiments are elaborately illustrated in Sect. 3.1, results are discussed in Sect. 3.2, followed by suggestions for improvement in Sect. 3.3. Finally, Sect. 4 concludes our work.

## State of the Art

Researchers have worked on technology to improve the experience of brainstorming meetings in particular for sighted people. Pictorial stimuli is used for supporting group conversation [22]. Graph-based web services are built for the solutions for various problems in meetings [6]. An automatic system to categorize and process the language used in meetings is described in [4]. Mobile phones are used for brainstorming sessions which act like a virtual mind map table [11]. There is also commercial as well as free tool support for brainstorming meetings. Approaches range from cards applications [5, 10] and mind map applications [12, 14] over dedicated brainstorming and decision support software [3, 21] to virtual design spaces and visual management tools [15, 20]. These various kinds of software allow for an improved workflow and help people to collaborate.

There is only little research to improve the integration of BVIP in brainstorming meetings. In [8], Mindmap-based brainstorming sessions are described to push the integration of BVIP in meetings. In [19], a Mindmap along with a LEAP sensor is described for tracking pointing gestures over an interactive horizontal surface. A prototypical system simulated gestures by sighted users and made them accessible to BVIP [16]. A system using a LEAP sensor and speech recognition was developed to improve the tabletop interaction for BVIP in [9] to better detect deictic gestures that are typically accompanied with specific words that hint to a geometric position. Another approach to detect pointing gestures in brainstorming meetings used a Kinect and a PixelSense table. It helped BVIP to understand the basic meaning of such gestures [7]. For this, an information infrastructure was developed by [17] to translate the natural behavior of sighted team members and thus reduce the information gap for the BVIP.

## Methodology

Our approach includes the development of a brainstorming tool and an autonomous system for recognizing pointing gestures. Thereafter, the two systems are combined to know the output of pointing gestures made towards the digital screen showing the brainstorming tool. This combined system helps BVIP to access the content of the brainstorming tool app, i.e. the card on which a sighted user is pointing to.

### Concept of the Brainstorming Tool

The brainstorming tool is software, which aims to support brainstorming meetings based on the Metaplan method. It mainly supports two different roles: a moderator and the other participants of the group. These participants can be sighted people as well as BVIP. The moderator organizes the input of the participants, leads the discussion, and asks participants to clarify and resolve input, but neither provides content nor makes decisions by himself. The participants on the other hand provide input by editing cards, and in a second step contribute to discussions and participate in the decision-making process. Consequently, the brainstorming tool has two different modes of operation, which will be used consecutively following the two different phases of Metaplan:

- Participants add cards via a smartphone Android application
- The moderator operates a web-based user interface, called whiteboard view, to organize cards of the participants

**Android App for the Participants.** The Android app for the participants has intentionally a relatively small feature set, since any detailed user interface would distract the user from his main task. The functionalities of the Android app are as follows:

- Participants can create cards and edit them.
- Providing an overview of all created cards by each individual user.
- Participants can submit cards to whiteboard. Once the card is submitted, it cannot be deleted anymore from the whiteboard by the participant.

**Web-Based User Interface for Moderators.** The web-based user interface for moderators includes the following functionalities for organizing and facilitating a meeting:

- Organization Moderators are provided with an overview of meetings. They can create new meetings, invite participants to a meeting from the list of users, who registered to the system, and can modify and delete existing meetings. Moderators can open meetings multiple times, which allows for multi-screen setups where screens show certain segments of the whole work space.
- Facilitation In the whiteboard view, moderators can rearrange cards, which were created by the other participants using the Android app. New cards pop up in real time on a stack in a corner of the virtual whiteboard. Moderators can create groups and relations between cards. However, they cannot decide to create these two types of entities themselves, but they are the output of group discussion. Moderators can delete cards, groups and relations. This is the result of a group discussion among participants coordinated by the moderator.

**Fig. 1.**
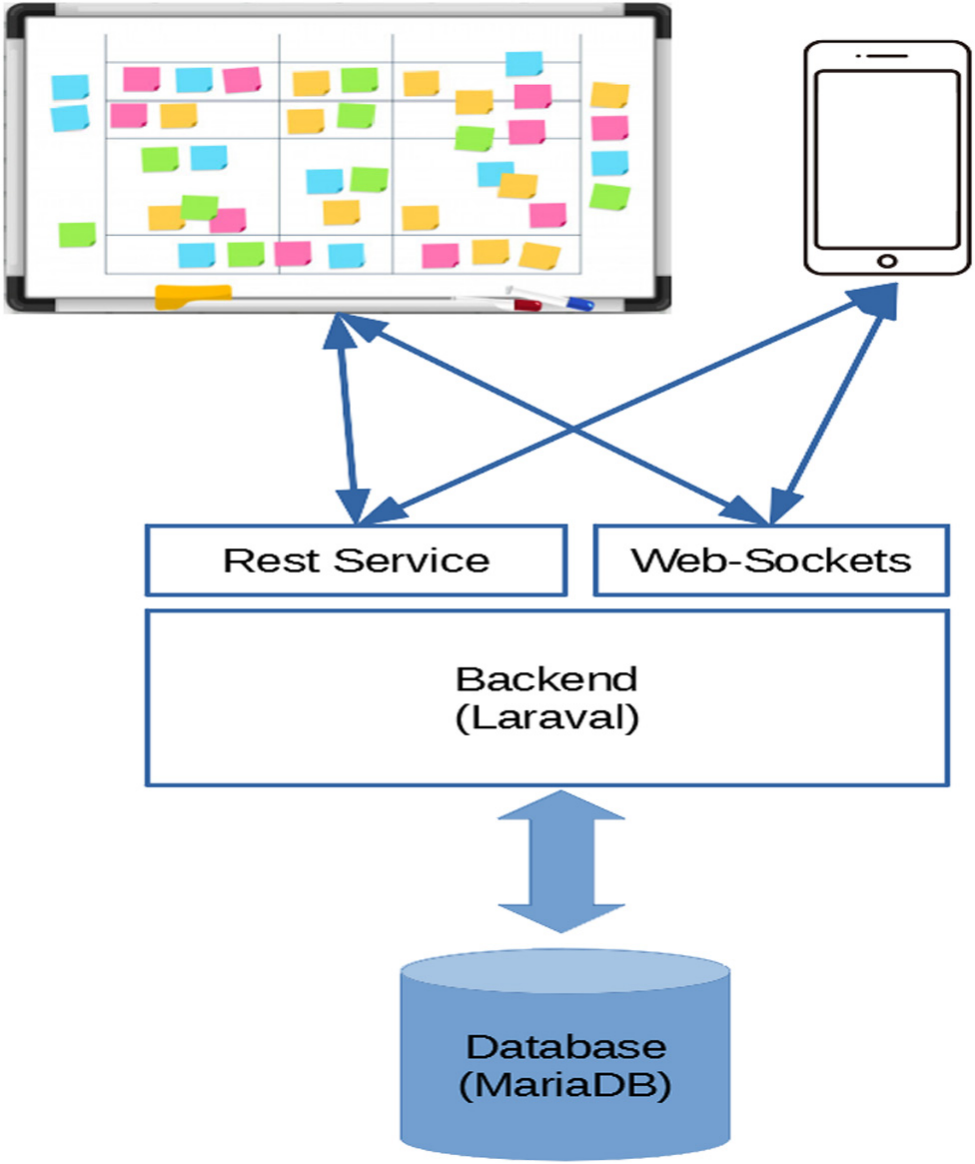
Components of the brainstorming tool.

**Architecture and Technology.** The brainstorming tool is based on a client-server architecture (see Fig. 1). The server is based on Laravel^1^ which stores data in an SQL database. Laravel also provides the web-based user interface for the moderator. For the dynamic parts of the whiteboard view, which are supposed to change without page reloads, like real-time modifications of the size, orientation and position of user interface elements or repositioning and grouping of cards, the JavaScript Framework Konva^2^ is used to display cards, groups of cards and their relation to each other. Konva allows the moderator to manipulate these items in a user-friendly manner using a mouse or touchscreen.

The server offers two kinds of APIs. Firstly, a RESTful API^3^, which allows data, e.g. user data, cards, groups, relations and other data, to be created, read, updated and deleted. Secondly, a Web-Socket^4^ service, which allows broadcasting changes of such data following the publish-subscribe pattern^5^. Clients can subscribe to channels, which correspond to sets of data. If a set of data changes, the server publishes the fact that data was changed to these channels, and clients can react to these changes and for instance update their cached data.

**Fig. 2.**
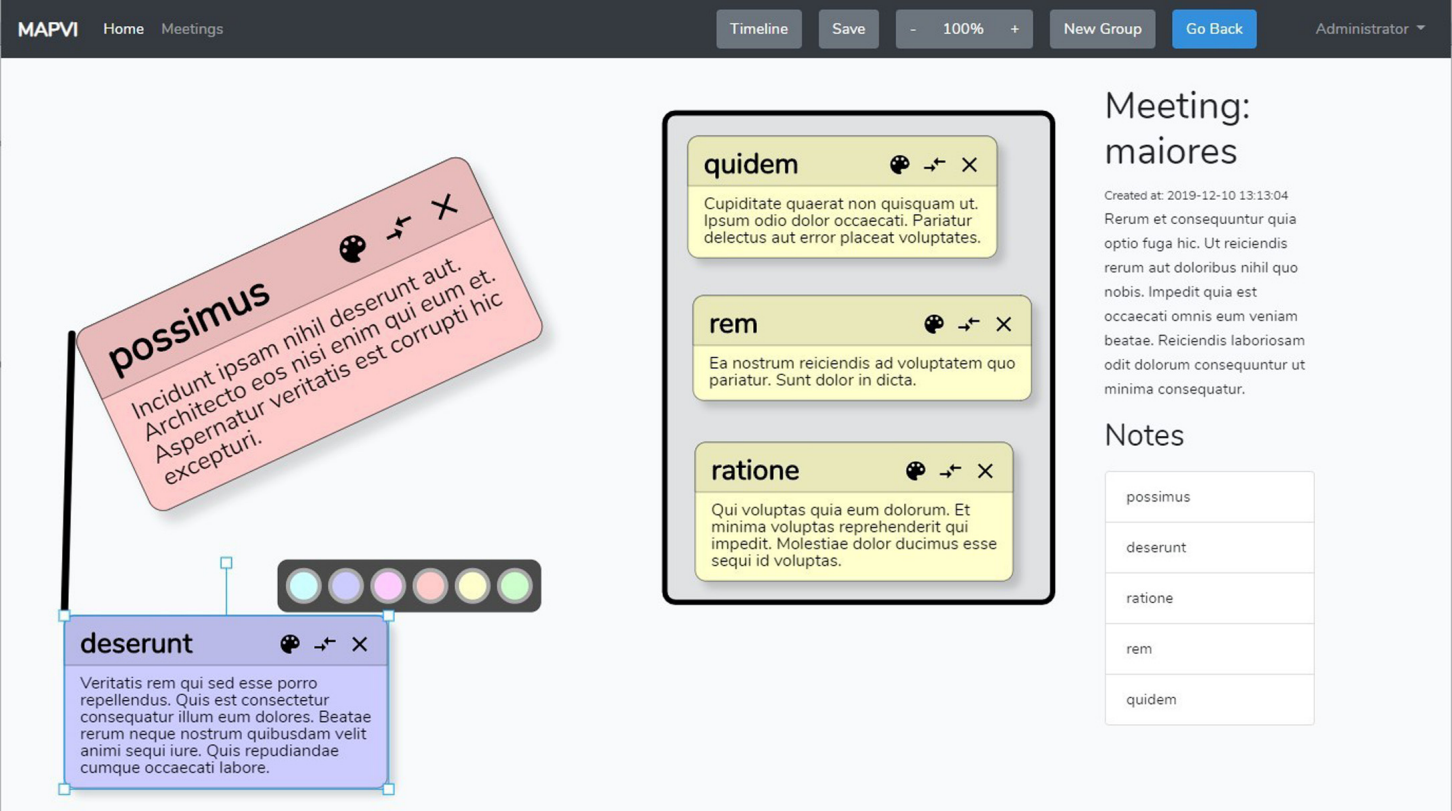
Screenshot of the brainstorming tool.

### Pointing Gesture Recognition System

The pointing gesture recognition system [2] uses a Kinect v2 sensor. The sensor data is given to ROS 1 (Robot Operation System) and analyzed by OpenPTrack [1] to get the joint coordinates of the pointing arm. These joint coordinates are then used for assessing the pointing gesture performed by the user. Each joint has a different ID and the x, y, z coordinates with different IDs are published. The sensor’s reference frame is transformed to the world reference frame using the /TF ROS package. This package is used for rotation and translation, i.e. linear transformations, to have the world reference coordinate frame.

The pointing gesture consists of an arm movement towards the referral object, and the hand pointing towards the object. The hand gesture is usually accompanied with speech referring towards the same directional position. We calculated the pointing gesture from the elbow and hand position coordinates. These coordinates help to find the forearm vector which is used for calculating the pointing vector. We used the mathematical transformation as shown in Eq. 1. For this, we used a normal direction to the plane $\textit{\textbf{N}}_f$, a predefined point on the ground plane $P_f$, the positions of hand *H*, and the position of elbow joint *E*, respectively.

1 $$\begin{aligned} P_p=H+\frac{(H-P_f)\cdot \textit{\textbf{N}}_f}{\textit{\textbf{EH}}\cdot \textit{\textbf{N}}_f}\cdot \textit{\textbf{EH}}, \end{aligned}$$

The plane coordinate frame is the plane where the output screen (the common work space for the Metaplan) is placed. The coordinate position in the world reference frame is transformed to the plane coordinate frame of the output screen using a rotation matrix. The output values from OpenPtrack are converted to the whiteboard/matrix plane coordinate frame. The TF package in ROS is used for this coordinate transformation. These transformed output position values are analysed based on the position of the cards of the brainstorming tool being displayed on the screen. After getting the position of the card being pointed at, the card’s content could be converted to speech and made available to the BVIP (Fig. 2).

**Fig. 3.**
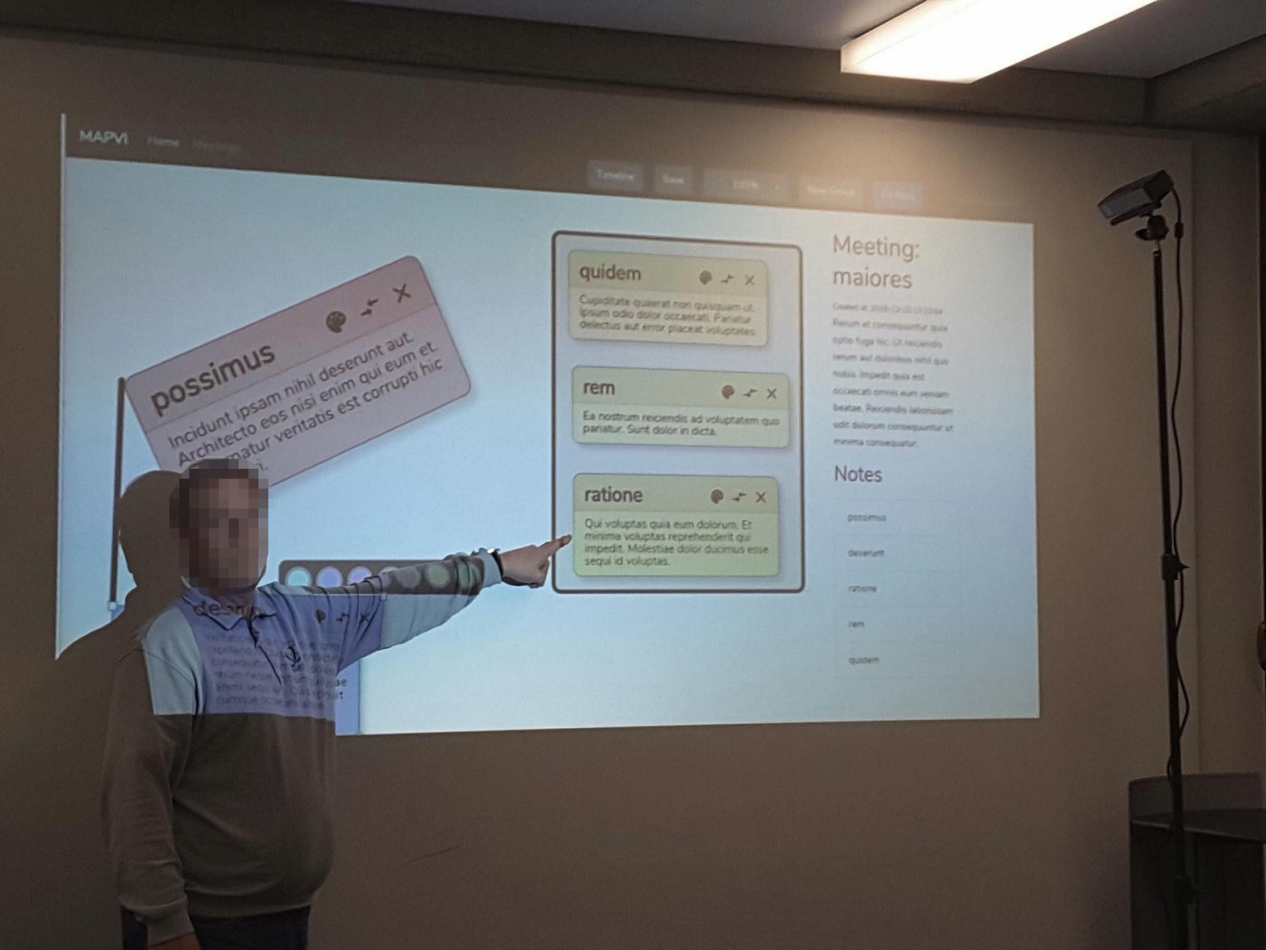
Illustration of a user pointing at the digital screen with the brainstorming tool and RGB-D sensor is used for capturing pointing gesture.

### Combination of Brainstorming Tool and Pointing Gesture Recognition System

After developing the brainstorming tool and the pointing gesture recognition system, these two systems are combined to better integrate BVIP in brainstorming meetings as shown in Fig. 3. The pointing gesture recognition system is used to assess the position of the card which is being pointed at by the moderator. This card carries the information which has to be conveyed to the BVIP. The system helps a BVIP to be better integrated and to access complete meaning of the conversation by knowing the contents the participants are talking about. So, it is a two-fold process: (1) The user points at the digital whiteboard where the contents of the web application of the brainstorming tool is displayed. The pointing gesture recognition system identifies the gesture and the target position of the pointing gesture. (2) The identified position is correlated to the content being displayed on the screen at that time to retrieve the contents of the corresponding artifact. Preliminary user studies on a screen with six equally distributed areas, this combined setup can offer 80 % accuracy in detecting the target position of pointing gesture.

## Conclusion

We built a brainstorming tool and automatic pointing gesture recognition system, which can work together in an synchronous manner to help BVIP to access the integral meaning of NVC. The output of our system could be delivered to the BVIP via audio/speech or using a braille display.

The pointing gesture recognition system is based on the pre-developed software OpenPtrack and ROS. The output of the system gives the position of the pointing gesture towards the digital screen showing the web application of the brainstorming tool. Future work will also involve the output medium for the BVIP. We plan to use a magnetically driven 2D actuation system along with braille display and audio for the output of the system.

## Footnotes

1 Laravel - PHP web framework: https://laravel.com.

2 Konva - Javascript 2D Canvas Library: https://konvajs.org.

3 REST: https://en.wikipedia.org/wiki/Representational_state_transfer.

4 WebSocket: https://en.wikipedia.org/wiki/WebSocket.

5 https://en.wikipedia.org/wiki/Publish-subscribe_pattern.
